# Prisoners co-infected with tuberculosis and HIV: a systematic review

**DOI:** 10.7448/IAS.19.1.20960

**Published:** 2016-11-15

**Authors:** Chantal L Edge, Emma J King, Kate Dolan, Martin McKee

**Affiliations:** 1 London School of Hygiene and Tropical Medicine, London, England; 2 Brighton and Hove City Council, Brighton and Hove, England; 3 Program of International Research and Training, National Drug and Alcohol Research Centre, UNSW Medicine, University of New South Wales, Sydney, Australia; 4 ECOHOST, London School of Hygiene and Tropical Medicine, London, England

**Keywords:** HIV, tuberculosis, TB, co-infection, prisoners, outcomes, incidence, prevalence

## Abstract

**Introduction:**

Almost from the beginning of the HIV epidemic in 1981, an association with tuberculosis (TB) was recognized. This association between HIV and TB co-infection has been particularly evident amongst prisoners. However, despite this, few studies of TB in prisons have stratified results by HIV status. Given the high prevalence of HIV-positive persons and TB-infected persons in prisons and the documented risk of TB in those infected with HIV, it is of interest to determine how co-infection varies amongst prison populations worldwide. For this reason we have undertaken a systematic review of studies of co-infected prisoners to determine the incidence and/or prevalence of HIV/TB co-infection in prisons, as well as outcomes in this group, measured as treatment success or death.

**Methods:**

A literature search was undertaken using the online databases PubMed, Embase, IBSS, Scopus, Web of Science, Global Health and CINAHL Plus. No restrictions were set on language or publication date for article retrieval, with articles included if indexed up to 18 October 2015. A total of 1975 non-duplicate papers were identified. For treatment and outcome data all eligible papers were appraised for inclusion; for incidence/prevalence estimates papers published prior to 2000 were excluded from full text review. After full text appraisal, 46 papers were selected for inclusion in the review, 41 for incidence/prevalence estimates and nine for outcomes data, with four papers providing evidence for both outcomes and prevalence/incidence.

**Results:**

Very few studies estimated the incidence of TB in HIV positive prisoners, with most simply reporting prevalence of co-infection. Co-infection is rarely explicitly measured, with studies simply reporting HIV status in prisoners with TB, or a cross-sectional survey of TB prevalence amongst prisoners with HIV. Estimates of co-infection prevalence ranged from 2.4 to 73.1% and relative risks for one, given the other, ranged from 2.0 to 10.75, although some studies reported no significant association between HIV and TB. Few studies provided a comparison with the risk of co-infection in the general population.

**Conclusions:**

Prisoners infected with HIV are at high risk of developing TB. However, the magnitude of risk varies between different prisons and countries. There is little evidence on treatment outcomes in co-infected prisoners, and the existing evidence is conflicting in regards to HIV status influence on prisoner treatment outcomes.

PROSPERO Number: CRD42016034068

## Introduction

Almost from the beginning of the AIDS epidemic in 1981, an association with tuberculosis (TB) was recognized. This relationship is attributed both to the suppression of host immune systems by human immunodeficiency virus (HIV) [1] as well as environmental risk factors shared by the two diseases [2]. People infected with HIV are 20 to 30 times more likely to develop active TB than HIV-negative persons, with an estimated one in three deaths among HIV-positive persons due to TB in 2014 [3]. Although deaths from TB in those living with HIV have fallen throughout the last decade, 360,000 deaths were attributed to co-infection in 2013 [4].

This association between HIV and TB co-infection has been particularly evident amongst prisoners. Between 1976 and 1986 there was a fivefold increase in the incidence of TB among inmates in the New York prison system, attributed in part to infection with HIV [5]. Studies in other high-risk populations such as intravenous drug users added to evidence linking the two conditions [6], as did a 1990 study of the prison population in Maryland that documented an increased risk of infection with TB among prisoners who were HIV positive. This increased risk fell just short of significance, but this study may have lacked adequate power to detect a difference [7].

There are several reasons for this association. First, immunosuppression from HIV infection both predisposes individuals to TB reactivation [8,9] and increases the risk of progression from infection to disease [8,10]. Second, there is some limited evidence from the USA that prisoners with a history of TB may be more likely to engage in behaviours that place them at a high risk of contracting HIV [11]. Third, the two infections share a number of socio-demographic and behavioural risk factors with each other and with the probability of incarceration, such as injecting drug use [2]. Fourth, conditions in prisons, such as poor ventilation and overcrowding, increase the risk of transmission of TB. Fifth, racial and ethnic groups that tend to experience proportionately higher TB rates, such as blacks and Hispanics in the USA, are often vastly over-represented, by up to 30 to 40 times [12], within prison populations [13–15]. Yet, despite the coalescence of all of these factors, knowledge of the epidemiology of HIV/TB co-infection among those who are incarcerated remains fragmentary.

There is already a large body of empirical evidence showing that history of imprisonment is an important risk factor for TB among those with AIDS [16,17] and those who engage in high-risk behaviours for HIV infection [18]. However, co-infection with HIV and TB among prisoners poses risks not only to the prisoners themselves, but also to prison staff [19,20] and contacts following release.

Risks associated with imprisonment extend beyond the prison walls. Numerous studies have documented TB increases in the broader population that can be attributed, at least in part, to contacts with prison populations [21–23]. More recently, studies using genetic epidemiology have provided important new insights into the role that prisons play in community outbreaks of TB [24,25].

In part, this is because prisons act as incubators of TB [26]. Research in Eastern Europe and the former Soviet Union has documented a close association between rates of incarceration and of both TB and multidrug resistant TB (MDR-TB), an association exacerbated by a high prevalence of HIV [27]. The aforementioned Russian study [25] found that TB spoligotypes (a means of genotyping *Mycobacterium tuberculosis* based on patterns of repeat units in DNA) were much less heterogeneous amongst prisoners than the general population, homeless or HIV-infected groups outside prison, suggesting that TB infection is transmitted especially easily amongst this confined group. Research in 1999 documented cases of HIV-positive prisoners co-infected with more than one *M. tuberculosis* strain [28].

Imprisonment also offers increased potential for TB outbreaks to run rife amongst closely confined inhabitants and prison staff, with numerous TB outbreaks having been recorded amongst correctional facilities in the USA. In 1991 a New York State correctional facility experienced a MDR-TB outbreak from January to November 1991. Eight persons were identified as having MDR-TB, seven of whom were HIV-positive inmates and one a correctional facility worker, immunocompromised as a result of recent chemotherapy [29]. In South Carolina (1999 to 2000) contact tracing of 323 prisoners housed in the same dormitory as a TB index case, found 31 HIV-positive inmates infected with TB, alongside a medical student who examined the source case [30]. Meanwhile, between 1995 and 1996, TB outbreaks occurred amongst HIV housing units in two separate Californian correctional facilities. In prison A, a 500-person HIV housing unit, 14 inmates and the wife of the index case were diagnosed with drug-susceptible TB. In prison B, a 180-person HIV housing unit, an index case resulted in 15 further cases of TB amongst inmates [31].

Given the high prevalence of HIV-positive persons and TB infections in prisons [32–34] and the documented risk of TBin those infected with HIV, it is of interest to determine how co-infection varies amongst prison populations worldwide. In 2005 it was estimated that around one-third of co-infected persons in Central Asia were found in penitentiaries [35]. However, definitive estimates of the prevalence and incidence of co-infection in prisoners worldwide remains sparse, with evidence suggesting that prisons often fail to implement simple case-finding and screening procedures for infectious disease [36]. For this reason we have undertaken a systematic review of studies of co-infected prisoners, to determine the incidence and/or prevalence of HIV/TB co-infection in prisons, as well as outcomes in this group, measured as treatment success or death. In this review, the terms *prisoners* and *inmates* are used interchangeably and refer to both convicted and pretrial (on remand) persons held in prisons, jails, detention and other penal institutions.

## Methods

A literature search was undertaken the online databases PubMed, Embase, IBSS, Scopus, Web of Science, Global Health (GH) and CINAHL Plus. No restrictions were set on language or publication date, with articles included if indexed up to 18 October 2015. Medical subject and text word searches were combined for terms relating to HIV/AIDS, TB, prisoner/prisons and detention centres. Scopus searches were performed on title and abstract for all terms, IBSS searches on keywords and Web of Science searches as topics. The full search strategy including all terms used is in Appendix.

This resulted in 3791 papers, and 2016 remained after duplicates were removed by EndNote. All further library management was undertaken using EndNote software. Further duplicates were found when reviewing paper titles and abstracts. These were manually removed, leaving 1975 papers. Titles and abstracts from these papers were reviewed by two independent reviewers and were selected for further appraisal according to the following criteria for inclusion: data must be from empirical research; data must relate to either incidence and prevalence of co-infection *or* outcomes of co-infection; diagnostic methods employed in HIV and TB detection must be specified, including HIV antibodies and TB skin testing (TST) or direct microscopy but not x-ray alone; research must be undertaken in a prison, jail, pretrial detention centre or compulsory drug detention centre; data must be available on the population denominator; and information must be available on the timing of the study. Case definition for TB can include latent TB infection (LTBI) and active TB.

**Figure 1:**
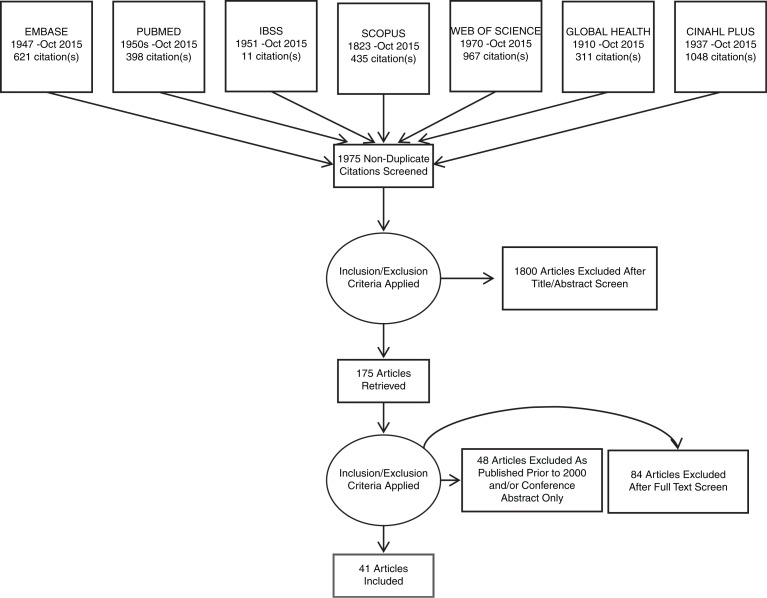
PRISMA flow diagram for articles on incidence and/or prevalence of HIV/TB co-infection in prisoners.

**Figure 2:**
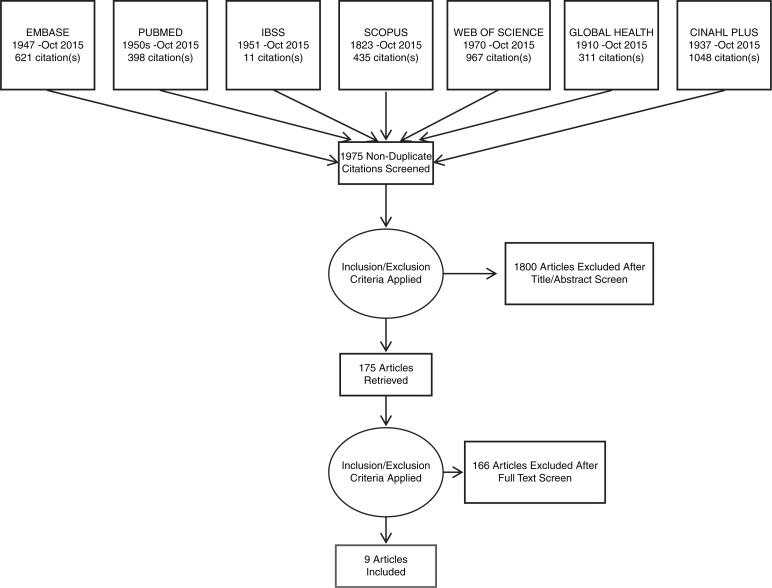
PRISMA flow diagram for articles on outcomes of HIV/TB co-infection in prisoners.

After papers that failed to meet inclusion criteria were removed, 175 papers remained for full appraisal and the full text was retrieved. After the full text appraisal, 46 papers were selected for inclusion in the review, 41 for incidence and prevalence estimates and nine for outcomes data, with four in both categories. For full details of paper selection, see Figures 1 and 2.

## Results

Table 1 presents illustrative examples of global research on TB/HIV co-infection. These studies provide further evidence that prisoners infected with HIV are at high risk of developing TB. However, the magnitude of risk varies between different prisons and countries.

**Table 1:** Studies of HIV/TB co-infection in prisons and its determinants

Country	Year of publication	Study population	Study method	Findings
Botswana [37]	2003	One prison, 1027/1173 prisoners participated	Point prevalence survey: Prisoners screened for TB, with some subsequently assessed for HIV status.	Of participants, 39 (4%) had TB; 14 declined voluntary counselling and testing, including three who reported previously testing HIV positive. Of the 20 prisoners with results available, 6 (30%) were HIV positive.
Brazil [38]	2013	Two prisons (PI and PII): 1351 samples in PI (23.9% of prison population) 2494 samples in PII (19.3% of prison population)	Retrospective study of diagnostic records. Samples were analyzed for both TB and HIV.	5/1351 examined for HIV and TB in PI were co-infected (prevalence of 0.4%; 95% CI 0.1 to 0.9). 11/2494 examined in PII were co-infected (prevalence of 0.4%; 95% CI 0.2 to 0.8). OR 0.84 (95% CI 0.25 to 2.61, *p*=0.744). 5/38 (13.2%) inmates with TB in PI were co-infected with HIV. 11/119 (9.2%) of TB cases in PII were co-infected (*p*=0.487).
Brazil [39]	2004	Four jails, 4293 prisoners	Retrospective analysis of TB cases in inmates between 1993 and 2000.	Among patients analyzed in the second year of treatment (*n*=359), HIV status was known in 73.6% of prisoners. Of these 67.8% were co-infected with HIV. AIDS was the most commonly associated disease with TB, at 49.9%.
Brazil [40]	2013	249/1261 prisoners participated (jail)	Cross-sectional study of prisoners to estimate active and latent TB prevalence using TST screening.	In a Poisson regression, HIV positivity was not significantly associated with latent TB infection (prevalence ratio 0.40, 95% CI 0.0558 to 2.8662, *p*=0.3618).
Brazil [41]	2009	237 hospitalized prisoners	Cross-sectional study of prisoners in hospital. Prisoners screened for TB symptoms.	6/237 (2.5%) diagnosed with active TB and 156 further tested with TST, showing latent TB prevalence of 61.5% (96/156). 87/237 prisoners submitted to HIV serology – all HIV negative.
Brazil [42]	2005	1081/1171 prisoners participated	Prisoners screened for TB.	43 cases of TB identified. 14.6% (6/41) HIV prevalence amongst 41 TB cases tested (2/43 TB cases refused HIV testing). Compared to 1.6% (15/950) prevalence of HIV in TB-negative prisoners. HIV positivity was significantly different between those with/without TB (*p*<0.0001).
Brazil [43]	2015	304/764 prisoners participated	Prisoners screened for TB symptoms (from a combination of active and passive case finding).	18% (7/36) of TB patients were HIV positive. In multivariate analysis, HIV positivity was not predictive of TB infection (PR 1.57, 95% CI 0.47 to 6.48, *p*=0.397).
Cameroon [44]	2006	2474/2830 prisoners participated	Prisoners screened for TB.	HIV seroprevalence in prisoners without clinical signs of pulmonary TB was 111/1067 (10.4%) but 6/24 (25%) in those with disease.
Cameroon [45]	2011	40/3219 prisoners participated	Prisoners screened for PTB, and HIV status reported for positive PTB cases.	4/40 (10%) newly positive PTB prisoners were HIV positive. 197/3179 (7.8%) prisoners without PTB were HIV positive (*p*=0.83). In 93 prevalent cases of PTB, 14/93 were HIV positive (15.1%, 95% CI 8.8 to 23.4), compared to 7.8% in general prison population (*p =*0.003).
Colombia [46]	2013	1305 prisoners with respiratory symptoms	Prisoners screened (4463/9507), and those with respiratory symptoms (1305/9507) tested for TB.	72 diagnosed with TB. 3/72 (4.2%) had co-infection. 23/1233 (1.9%) without TB were HIV positive.
Colombia [47]	2012	2103 prisoners with respiratory symptoms and their contacts (jail)	Prospective cohort study for TB.	Two cases of co-infection found.
Colombia [48]	2015	72 prisoners with TB	Retrospective analysis of TB cases.	Co-infection in 11 (12.4%) cases; although 36 (37.4%) had no HIV status information, 25 (37.4%) were known HIV negative.
Ethiopia [49]	2012	250 prisoners with cough >1 week	Those with cough lasting >1 week assessed for TB and HIV status.	26 with TB, 19 HIV positive (73.1%), of whom 9 had co-infection. 20 of those with TB developed cough after incarceration.
Iran [50]	2015	2931 patients with TB	291 prisoners with TB, compared to 2640 TB cases in general population.	9 (2.4%) prisoners co-infected with TB, compared with 35 (1.3%) in general population (*p*=0.025). However, only a minority of prisoners were tested for HIV.
Kenya [51]	2010	144 prisoners	Case-control study. Cases: 48 smear-positive prisoners. Controls: 96 with no cough or recent treatment.	Risk factors for TB. Self-reported HIV-positive status OR 10.75 (95% CI 2.42 to 47.77). Contact with the case of pulmonary TB OR 6.68 (95% CI 1.17 to 38.23).
Malaysia [52]	2013	266/1400 prisoners participated	Cross-sectional survey of prisoners screening for TB and HIV status.	TST reactivity significantly lower in those who were HIV positive but prevalence of TB symptoms similar (16.9% in HIV positive and 10.1% in HIV negative, *p=*0.105). Odds ratio for TB in those who are HIV positive 1.82 (95% CI 0.88 to 3.76).
Malaysia [53]	2013	143 prisoners with HIV	Prisoners with HIV assessed for TB prevalence.	Active TB prevalence of 16.7% (24/143) amongst HIV-positive prisoners (165 times higher than the Malaysian general population) – study revealed 12% (15/125) of HIV-positive prisoners had undiagnosed active TB at screening.
Mexico [54]	2006	132 prisoners with TB (social readaptive correctional facility)	TB cases amongst all prisoners (132/5375) assessed for HIV status.	10/132 (7.58%) of TB cases in a correctional facility were HIV positive.
Mexico [55]	2012	172 prisoners with HIV	HIV-positive prisoners were assessed for TB infection.	28/172 (16.3%) were co-infected with TB (21/172 (12.2%) with pulmonary TB). Incidence rate of 7.7/100 persons/year for active TB and 4.7/100 persons/year for pulmonary TB.
Nigeria [56]	2010	168 prisoners	Sample of prisoners screened for TB and HIV status.	1/168 active TB and HIV positive, 25/168 LTBI and HIV positive. 24% (6/25) HIV-positive inmates with positive TST developed active TB. Compared to 13.8% (8/58) in HIV-negative inmates.
Nigeria [57]	2009	48 prisoners with TB	Retrospective study of TB cases in Kuje Prison, April 2004 to December 2008.	4.2% of TB cases were co-infected with HIV; all these patients died in treatment, whilst non-co-infected prisoners survived.
Russian Federation [2]	2005	1345 prisoners with TB.	Prisoners with TB assessed for HIV status.	12.2% co-infected with HIV, compared with 1.7% among those with TB in general population.
South Africa [58]	2014	981/1046 prisoners participated	Cross-sectional survey screening for TB and HIV status.	Of 968 declining treatment, 25.3% were HIV positive. Positive HIV status was independently associated with undiagnosed TB (OR 2.0, 95% CI 1.0 to 4.2).
South Africa [59]	2012	148 prisoners with HIV	All HIV-positive prisoners (148) attending an ART clinic between April 2004 and Feb 2008 were assessed for PTB.	At baseline, 69/148 prisoners had pulmonary TB and 14/148 extra-pulmonary TB.
South Africa [60]	2015	1140 prisoner records reviewed	Retrospective case control to review data from inmates diagnosed (cases) versus not diagnosed with TB (controls). 1040 non-TB and 100 TB cases.	58% (*n*=58) TB cases HIV positive, 27% (*n=*27) HIV negative, 15% (*n*=15) unknown HIV status. Compared to non-TB cases, 18.5% (*n*=192) HIV positive, 36.9% (*n*=384) HIV negative, 44.6% (*n*=463) unknown HIV status. Being HIV positive increased the odds of developing TB (OR 4.2, 95% CI 2.64 to 7.00).
South Africa [61]	2013	202 prisoners on TB treatment	Prisoners who commenced TB treatment between 2007 and 2010 assessed for HIV status.	109 (54%) HIV positive.
Spain [62]	2001	9 prisons 7524 prisoners	Prisons in Madrid area compared to general population of South Madrid (455,050 inhabitants).	304 culture-positive TB cases, 221 isolates typed (73 prisoners, 148 urban population). Prisoners: 69/73 TB cases with known HIV status; 49/69 (71.0%) had co-infection. General population: 98/148 TB cases with known HIV status; 37/98 (37.8%) had co-infection. Current imprisonment increased risk of inclusion in a TB cluster for HIV-negative cases (adjusted OR 6.3, 95% CI 1.8 to 21.8). Previous imprisonment increased risk of inclusion in a TB cluster for HIV-positive cases (OR 13.0, 95% CI 3.7 to 45.6).
Spain [63]	2000	97 prisoners with TB	Prisoners with TB assessed for HIV status.	71% of TB patients were HIV positive.
Spain [64]	2012	371 prisoners	Prisoners screened for TB and HIV.	HIV prevalence was 10.8% (95% CI 7.5 to 14 – 40 prisoners), of which 63.2% were co-infected with TB, representing 6.7% of total prison population co-infected.
Spain[65]	2001	1050 prisoners	Cohort study for TB incidence.	10% co-infected, with 23 cases of TB. Relative risk of TB in HIV-positive prisoners 4.07 (95% CI 2.61 to 6.35).
Tajikistan [66]	2014	Two prisons, 1317/1350 prisoners participated	Behavioural and demographic cross-sectional survey with radiographic screening for pulmonary TB.	12 (0.9%) HIV positive, of whom 4 (33.3%) had TB, compared with 55/1301 HIV negative.
Tanzania [67]	2001	501 prisoners with TB	Retrospective cohort study of prisoner case notes.	204 (40.7%) with smear-positive TB, of whom 25.9% had HIV co-infection.
Uganda [68]	2014	469 prisoners with TB	Prisoners with TB assessed for HIV status.	268/469 (57.1%) HIV positive, 192/469 (40.9%) HIV negative, 9 unknown.
Ukraine [69]	2008	203 prisoners with TB	Prisoners with TB assessed for HIV status.	165/203 (81.3%) HIV negative, 36/203 (17.7%) HIV positive, 2/203 (1.0%) unknown HIV status. MDR-TB was significantly associated with HIV positivity and incarceration in multivariate analysis (compared to HIV negativity and civilian populations).
Ukraine [70]	2008	156 prisoners with TB	Prisoners with newly diagnosed TB assessed for HIV status.	24/156 prisoners smear-positive TB (others not confirmed/unknown smear status); 5/24 (20.8%) co-infected with HIV. Compared with civilian population smear-positive TB and HIV co-infection 83/397 (20.9%).
United Kingdom [71]	2014	511 prisoners	Prisoners screened for TB and HIV.	No patients with co-infection (65 IGRA positive for TB but 0 HIV positive).
United States [72]	2007	469 prisoners with TB	Prisoners with TB assessed for HIV status.	16% HIV positive, 63% HIV negative, 22% HIV status unknown.
United States [73]	2005	441 prisoners with TB (jail)	Prisoners with TB assessed for HIV status.	23.1% TB cases also HIV positive. This is compared to non-TB controls (478), where 8.2% were HIV positive (chi-square 39.58, *p*<0.01). In multivariate logistic regression, HIV-positive status was predictive of TB infection (OR 3.07, *p*<0.01).
United States [74]	2003	199,399 prisoners 46 correctional facilities (6 jails accounting for 1.4% of inmates)	Prisoners given TST.	TST read in 99.3%. 11,814/33,653 had positive TST and known HIV status. 44.9% (1991/11,814) HIV positive. 17% of those with a positive TST and known HIV status were co-infected. TB prevalence in inmates with HIV was 744/100,000 screened, versus 34/100,000 for all other inmates. Amongst inmates with a known HIV status, TST was 4.2 times (95% CI 3.9 to 4.4, *p*<0.001) more likely to be positive in HIV-positive patients.
United States [15]	2002	149,684 prisoners	TB incidence in HIV-infected prisoners.	41 prisoners developed active TB. TB rate was 395/100,000 persons (95% CI 214 to 725) in HIV-positive inmates (*n*=10/2533) and 20/100,000 (95% CI 14 to 28) in HIV-negative inmates (*n*=29/147,151).
Zambia [23]	2013	2323/2514 prisoners participated	Prisoners screened and data collected on entry, during incarceration and on and after release.	Proportion of bacteria logically confirmed TB cases that are HIV positive: entry 5/14 (35.7%); in prison 22/48 (45.8%); exit 1/2 (50%); prison camp community 7/7 (100%). Proportion of HIV-positive persons with confirmed TB entry 5/64 (7.8%), in prison 22/342 (6.4%), exit 1/12 (34.3%), community 7/57 (24.6).
Zambia [75]	2014	1853 prisoners	Prisoners screened for TB and HIV.	366 inmates excluded, including 52 inmates (2.8%) already on TB treatment. Of the remaining 1487 inmates, 62/1487 (4.2%) were TB positive, of which 27/62 (43.6%) were also HIV infected. Compared to 24.1% (343/1425) non-TB patients with HIV infection, *p*=0.0005. Being HIV infected gives an OR of 2.4 (1.5 to 4.1, *p*=0.0007) for TB positivity.
Zambia [76]	2015	7638 prisoners	Prisoners screened for TB and HIV status assessed.	Of 7638 prisoners screened for TB, 4879 were also tested for HIV (625 already known HIV positive). HIV prevalence of 37% (49/132) in prisoners with bacteriologically positive TB, and 37% (80/214) among prisoners with clinically diagnosed TB.

ART, antiretroviral therapy; CI, confidence interval; LTBI, latent tuberculosis infection; MDR-TB, multidrug-resistant tuberculosis; OR, odds ratio; TB, tuberculosis; TST, tuberculin skin testing; IGRA, interferon gamma release assay; PTB, pulmonary tuberculosis.

Few studies reported the incidence of TB amongst HIV positive inmates[15,55,56,65], with most simply reporting results from cross-sectional surveys of TB/HIV prevalence or assessing HIV status amongst those that develop TB, as opposed to observing the incidence of TB amongst prisoners of known HIV status. Of the four papers that did provide measures of incidence, three found incidence of TB greater amongst those who were HIV positive [15,56,65], whilst one did not provide a comparison between HIV-negative and -positive prisoners [55]. This lack of reported incidence measures may be reflective of the challenges of calculating these rates in a dynamic, transient prison population, such as issues with accurate calculation of person time at risk, and exclusion of prevalent TB cases, which may or may not be apparent on entry [77].

Estimates of co-infection prevalence, as mentioned previously, tended to favour measuring HIV prevalence amongst TB cases, although some studies measured prevalence of TB in HIV-positive prisoners. Amongst TB cases, prevalence of HIV positivity varied from 0.1% [47] to 73.1% [49]. There were few clear geographical patterns within the results; studies performed in African countries often reported co-infection prevalence over 50% in TB cases [49,60,61,68]. As with measures of incidence, this wide variation in prevalence estimates may be in part due to the settings and characteristics of the prisoners themselves but also in part to methodological differences in calculation of prevalence.

Measures of association between TB and HIV positivity were variable amongst those studies that reported it, with some finding no significant association [40,58,60,65,73,75] whilst others reported highly significant associations between the two infections, with relative risks of co-infection ranging from 2.0 to 10.75 [51,58].

There was a high risk of bias in many of the studies included (Table A1), most often because studies were performed in those with TB or HIV, as opposed to the entire prison population. Few studies explicitly set out to measure co-infection, often including HIV positivity in a list of characteristics common to patients with TB. Even in studies that did not specify whether an infected group was targeted, most often a subset of the prison population was used, such as those with respiratory symptoms. Amongst the studies case definitions varied. In total, two examined LTBI diagnoses only, six at both active TB and LTBI and 35 at active TB only. The bias inherent in many of these studies means it is hard to draw any definitive conclusions as to the size of the association or increased risk between TB and HIV in prisoners. For this reason a formal meta-analysis was not undertaken on the retrieved results, nor simple summary measures produced, due to the considerable heterogeneity between study methods and results as demonstrated in Table A2.

Only six of the studies retrieved [2,50,53,62,69,70] provided any comparison of a prisoner's risk of co-infection to that of the general population. All of these studies found the risk or prevalence of co-infection to be greater among prison residents than that of the general community. However, given the small numbers of studies reporting these comparative data, it is hard to ascertain a definitive value of increased risk. Therefore, we must simply conclude that the risk of developing TB amongst HIV-positive inmates is high and appears higher than for those outside of prison.

Few studies retrieved were recorded as performed in jails (used to hold individuals awaiting trial or sentencing) [39,40,47,73,74], meaning most of the conclusions drawn were based on those resident in prisons, who are likely to be less transient than jail or remand populations. One jail study found HIV positivity predictive of TB (odds ratio (OR) 3.07; *p*<0.01) [73], whilst another found very few cases of co-infection (n=2/2103) [47], making it hard to draw conclusions on co-infection risk in jail. Those in jails may potentially be more likely to represent people who are incarcerated with TB, as short length of stay may preclude TB development; however this association is unproven. In addition, short-stay prisoners may represent a population with high rates of recidivism [78], therefore returning as a prevalent case if TB treatment completion was complicated by the short stay nature of jails [73].

While noting the caveats concerning TST, research suggests that prisoners are no more likely to be infected with TB [79] but are more likely to develop active disease [44,56,60,73] than people residing outside of prison. Indeed, one study found no association between HIV positivity in prisoners and LTBI [40]. One of the few studies among female prisoners, from Brazil, found that while the TB infection prevalence was similar among women with or without HIV infection, with a purified protein derivative test conversion rate of approximately 30% in both, the incidence of TB was 9.9 per 100 person-years among those who were HIV positive compared with 0.7 per 100 person-years in those who were negative [80]. This same study found that the development of TB was most likely within the first 12 months of incarceration, as was also the case in a Texan prison [15].

Some studies looking at activation of disease have found an association between TB and CD4 counts below 200 cells/mm^3^ [81,82], whilst others suggest a significantly increased risk below 350 cells/mm^3^, with risk rapidly increasing as CD4 count decreases [16]. Therefore, differing rates of active disease co-infection between settings may in part be explained by the stage of AIDS in HIV-positive prisoners. Receipt of antiretroviral therapy (ART) by prisoners could also impact on the observed heterogeneity in co-infection rates, with those receiving therapy less likely to succumb to opportunistic infections.

### Prevention and treatment

Low rates of TB treatment completion amongst the prison population in general are well documented, and the reasons are multifactorial. Many low- and middle-income countries [83] have severely limited prison health facilities, and prisoners with symptoms suggestive of TB may go undiagnosed. In addition, access to appropriate chemoprophylaxis in prisons can be problematic due to reasons such as affordability, circulation of suboptimal drugs and the low priority given to prison healthcare by policymakers [84,85]. In addition, the movement of prisoners, both within and out of the prison system, exacerbates the rate of treatment default.

Evidence on the quality and effectiveness of treatment in those with co-infection is fragmentary. However, the available evidence indicates that, even in general populations, there are substantial geographical variations. For example, patients in Eastern Europe are significantly less likely to receive adequate treatment for both conditions than those in other parts of Europe or Latin America [86]. For prisoners with co-infection, treatment completion is complicated by both the nature of this transient population and the complex drug regimes required for co-infected patients, sometimes resulting in adverse drug-drug interactions [15,87]. HIV positivity is not generally associated with TB treatment adherence [88]. However, one US study of prisoners with latent TB found that they were less likely to complete treatment if they were HIV positive, with defaults attributed to movements within the correctional system, refusal of treatment and adverse drug effects [74]. The results for treatment outcomes have been summarized in Table 2.

**Table 2:** Outcomes of prisoners with HIV/TB co-infection

Country	Study population	Method	Treatment default/outcomes	Association of outcomes with HIV positivity
Uganda [68]	469 prisoners with TB	Retrospective study of treatment completion	Default 12% amongst those staying in same prison; 53% for those transferred to another prison; 81% lost to follow-up once left prison.	HIV positivity not significantly associated with treatment default compared to HIV-negative patients (crude OR 0.88, 95% CI 0.61 to 1.28, adjusted OR 0.82, 95% CI 0.50 to 1.33).
Spain [88]	52 prisoners taking TB treatment	Compliance with treatment observed	Abandonment of TB treatment by HIV-positive people may pose a particularly high threat of developing TB resistance or relapses.	HIV positivity not significantly associated with treatment default (*p*=0.86).
Spain [89]	62 patients (46 HIV positive, 16 HIV negative)	DOTS adherence after release		HIV positivity not significantly associated with treatment outcomes (OR 1.94, 95% CI 0.14 to 18.95, *p*=0.40).
USA [74]	Prisoners with latent TB	Compliance with treatment observed	14.2% defaults attributed to movements of prisoners, 5.2% to other loss to follow-up, 3.6% to treatment refusal, 2.9% to adverse drug effects.	HIV positivity associated with failure to complete 12-month treatment regime (40%) compared to HIV-negative patients (68.1%) (OR 0.24, 95% CI 0.20 to 0.28, *p*<0.001).
South Africa [59]	148 prisoners on ART	Retrospective cohort study: Outcomes of HIV-positive prisoners referred to an ART clinic over four years	Most common reason for hospitalization of prisoners on ART was TB (17/45 or 38%). 44/133 (33%) prisoners were on TB treatment, at ART initiation. New infections with both PTB/extra-pulmonary TB occurred equally before, during and once established on ART treatment. By study end 21% (31/148) of prisoners on ART had received medication for a new TB episode. 5/8 prisoners who commenced ART ≤1 month of starting TB treatment developed immune reconstitution inflammatory syndrome. Ultimately, prisoners on ART had good clinical, virological and immunological outcomes despite high TB incidence.	
Mexico [55]	28 cases TB amongst 172 HIV-positive prisoners	Cohort study of HIV-positive prisoners	Of 28, 20 completed, 3 died and 5 did not attend treatment post-release. 23 patients received concomitant treatment for HIV/TB – 9/23 developed immune reconstitution inflammatory syndrome.	
USA [90]	10 co-infected prisoners	Review of patient case notes	All patients died before treatment completion.	
USA [91]	225 patients receiving LTBI treatment (222 HIV positive, 3 HIV negative)	Observation of outcomes amongst prisoners receiving anti-TB medication during a TB outbreak in an HIV-infected housing unit	158/225 (70%) completed DOTS in prison; 19/225 (8%) released prior to treatment completion. 24/225 (11%) had treatment discontinued due to adverse effects; 13/225 (6%) later admitted non-adherence to the regime even though they had received DOTS. 13 prisoners with HIV were treated with rifabutin regimes. 7/13 (54%) completed treatment in prison, whilst 5/13 (38%) were released before completion.	
South Africa [61]	202 prisoners with TB	Retrospective review of case notes	HIV-positive prisoners achieved a TB cure rate of only 40%, compared to the overall rate of 47%, although whether these numbers were significantly different was not reported.	Poor treatment outcomes (death, treatment failure and relapses of TB after successful treatment) associated with HIV positivity. Crude OR (OR 3.79, 95% CI 1.35 to 10.23) did not adjust for factors such as transfer out of prison.

ART, antiretroviral therapy; CI, confidence interval; DOTS, directly observed treatment, short course; LTBI, latent tuberculosis infection; OR, odds ratio; TB, tuberculosis.

There have been a few studies of TB outbreaks among HIV-positive prisoners. One review concluded that, although consideration must be given to drug-drug interactions involving ART and anti-TB medication, shorter drug regimes remain preferable, due to the transitory nature of the prison population [91].

The available evidence provides several other important findings. First, in HIV-positive prisoners found to have evidence of infection by TST, latent TB infection treatment can reduce the incidence of TB [65]. Second, where effective treatment is provided, outcomes of TB infection among those infected with HIV, at least where immunosuppression is mild or ART is also provided, are as good as in HIV-negative prisoners [92], consistent with other evidence that treatment for drug-sensitive TB is effective in the early stages of immunodeficiency [93]. Third, in the presence of sensitivity testing and continuity of treatment, those with HIV infection are at no greater risk of developing MDR-TB, beyond their risk for TB in general [94,95], and, in one Russian study, prisoners with co-infection were significantly less likely to develop resistance, although the explanation was unclear [96].

Isolation of infectious prisoners to prevent further spread is not consistently documented. Moreover, many of those isolated may not be receiving treatment, conditions are frequently appalling and these facilities may be used to hold uninfected juveniles thought to be at risk from older, violent prisoners elsewhere in the prison [97]. As mentioned previously, the segregation of prisoners into HIV-infected units provides an ideal opportunity for TB to spread rapidly amongst this vulnerable population [31]. Even in high-income countries there may be problems. For example, in US prisons, Hispanics are systematically less likely to be tested for HIV and TB than white prisoners [98].

## Discussion

This review provides evidence that individuals infected with HIV who reside in a prison setting are at high risk of developing active TB, with this risk varying vastly by factors such as prison location and prison population density. This is consistent with literature reporting higher rates of TB and HIV individually, amongst prison populations worldwide, as noted in a recent paper from which this systematic review arose [99]. It has summarized an extensive body of literature on its determinants and the responses to it. It is clear that, if services appropriate to the scale of the problem are to be developed, further research is needed to accurately quantify the burden of co-infection in prison populations today and the differences between and within countries, as well as the outcomes prisoners can expect during and after treatment. In this review most of the studies citing numbers of co-infected prisoners did not have this as their primary objective, more often recording HIV positivity as one of numerous variables in patients with TB, so that these estimates of prevalence/incidence are merely a by-product of different research objectives. As noted by Rieder *et al*., new prison entrants may be more likely to participate in screening processes, meaning that prevalence estimates may be higher if made at a time when the proportion of new entrants was high. Prevalence can also vary according to the case definition used for inclusion [77]. With most studies reporting only cross-sectional results, it remains hard to disentangle whether imprisonment increases the risk of developing TB over the course of imprisonment, whether those with TB are more likely to be imprisoned and found to be HIV positive during prison-based screening, or whether in fact high prevalence estimates result from a combination of both these factors[A8]. Overall, it is apparent that there is a need for accurate quantification of the burden of co-infection in high-risk prison settings. Consideration must be given to the funding and administration in resource-poor settings. In England, which maintains a comprehensive infectious disease surveillance programme based in Public Health England, the national public health agency, there are problems in ascertaining rates of co-infection. Although [A9]TB is a notifiable disease and authorities are required to report it upon detection, the same is not true of HIV status, despite the associated benefits for public health, such as contact tracing and treatment [100]. Without robust surveillance systems to confirm numbers of co-infected prisoners, it will be nearly impossible to monitor and appraise the effectiveness of interventions to reduce the rates of co-infection.

Studies of treatment outcomes in those co-infected were even more limited, with results conflicting as to the impact of HIV positivity on treatment outcomes. Prisoners were often lost to follow-up post-release, thereby reinforcing the need for a robust continuum of care to ensure treatment completion as well as monitor and evaluate treatment effectiveness. Further research is needed to determine optimum treatment regimes, delivery models and expected outcomes in this population.

This reflects the relative paucity of studies on TB in prisons [101], few of which have stratified results by HIV status [102]. It also reflects the scarcity of studies of HIV/TB co-infection in general. A recent international systematic review of co-infection studies, excluding China, found 47 studies, and just one on prisoners [103]. However, this shed light on several important issues. First, those studies combining chest radiography with serology produced higher estimates, consistent with what is known about the lower sensitivity of sputum culture and the problems of interpreting TST in individuals who are immunosuppressed and therefore less likely to react [52,64,104,105]. Second, it confirmed an earlier study showing that co-infection was more likely in those communities where the prevalence of HIV was highest [106]. However, the use of different methodology precluded comparison.

Heterogeneity observed in study results is likely due in part to study design but also to the differences between prisons themselves. Prisons are diverse, both in terms of the accommodation style and the numbers and demographic characteristics of their inhabitants. Prisons may be mixed or single sex, with some previously segregating HIV-positive inmates into separate housing facilities, although this is now rare [31]. Rooms range from well-equipped single cells to cold, dark, damp concrete boxes accommodating large numbers of prisoners, with one study from Ethiopia reporting an average number of 333 prisoners per cell [49]. As one UN inspector visiting a predetention centre in Russia noted, it would need the literary skills of Dante or the artistic skills of Hieronymus Bosch, to describe fully the horrors with which she was confronted [107]. Recent data showed that, in 40% of 193 countries, prison occupancy was over 100% of stated prison occupancy capacity; more than half of overcrowded prisons had occupancy rates between 150 and 200% of capacity and 15 were above 200% of capacity. Some approached 400% [108,109]. A study that modelled the transmission dynamics of outbreaks of TB originating in institutions such as prisons and mines concluded that the most important factors were the size of the population within the institutions and movement between them and the community [110]. Therefore, it is almost inevitable that the scale of HIV/TB co-infection will vary between countries and, within them, between prison settings, regardless of study methodology and associated bias.

A recurring theme in the quest to improve treatment outcomes was loss to follow-up of patients released from prisons [61,74,89]. This topic has been the subject of numerous studies, particularly in regard to continuing HIV therapy [111,112]. A recent systematic review found improvements along the pathway of care for HIV during incarceration, often beyond what was achieved in the general population, but problems rapidly arose as soon as subjects were released from jail [111]. Recommendations to address this problem are complex [112,113], acknowledging the competing demands in prisoners’ lives once they are released back into the community. As re-incarceration has been known to reach up to 76% within five years of release [113], inadequate treatment of LTBI [74], which later manifests as active TB in patients with HIV, may result in the reintroduction of TB both into the community and back into the prison system. In addition, loss to follow-up and therefore non-compliance with the full TB treatment regime contributes to the development of drug-resistant strains of TB [114,115]. Particular attention must therefore be paid to developing models of treatment continuum that are acceptable to prisoners post-release.

This systematic review is subject to certain limitations. Although there was no restriction on publication date for paper retrieval, we did not include data on co-infection incidence and/or prevalence from papers published prior to 1999, as their relevance to the current situation is dubious given the changing epidemiology of TB and HIV since this period. Prevalence or numbers of co-infected prisoners documented only in conference abstracts were not presented, as it is unknown whether this information was subjected to critical peer review, and many of these abstracts failed to document clearly the methods of ascertaining infection status. Several high quality studies documented rates of HIV, TB and other infections amongst a specific prison population but failed to give any data on numbers with co-infection. It is plausible that direct contact with these authors may have yielded further information. However, there may be doubt about whether the results provided would be accurate if co-infection was not specifically recorded and reported during the original study. Many of the papers presented did not set out purposefully to record levels of co-infection, but instead measured numbers of HIV-positive people amongst TB cases, or vice versa, meaning prevalence estimates within the entire prison population could not be calculated. International comparisons must also be undertaken with care because of the very different nature of prisons, the analyses undertaken in these studies and the methods used to identify TB infection. Moreover, rates of co-infection will be influenced by what is happening in the general population, which also varies greatly.

## Conclusions

If the tide of HIV/TB co-infection in prisons is to be stemmed, there is a clear need to develop and implement specific, coordinated policies for active case-finding and treatment of co-infected prisoners, for both those in prison settings and those transitioning back into the community. This will bring benefits to the individuals concerned but also reduce the reservoir of disease, both within the prison population and the community at large. The difference in the care provided to prisoners and the general population, as well as the priority it achieves on the policy agenda, has long been recognized.

## Appendix

Literature search terms and methodology

The following medical subject (MeSH) terms were included as exploded terms for *HIV/AIDS* if present in the database: Human immunodeficiency virus (Embase, GH, CINAHL), human immunodeficiency virus infection (Embase), acquired immune deficiency syndrome (Embase, GH), AIDS related complex (Embase), HIV (PubMed), HIV infections (PubMed, GH, CINAHL), Acquired immunodeficiency syndrome (PubMed), Acquired immunodeficiency syndrome virus (PubMed), AIDS related complex (PubMed), AIDS related opportunistic infections (PubMed, CINAHL), HIV seropositivity (PubMed, CINAHL), HIV seroprevalence (PubMed), HIV 1 (PubMed, CINAHL), HIV 2 (PubMed), AIDS serodiagnosis (CINAHL). These terms were also combined with text word searches for the following: HIV infect* OR HIV OR HIV OR HIV-1* OR HIV-2* OR human immun#deficiency virus OR human immun#-deficiency virus OR acquired immun#deficiency syndrome OR acquired immun#-deficiency syndrome OR AIDS OR AIDS related complex OR AIDS related opportunistic infection* OR HIV seropositivity OR HIV seroprevalence OR HIV 1* OR HIV 2* OR HIV/AIDS OR HIV-infected. Searches in Scopus were performed on title and abstract for all terms, and as topics in Web of Science. IBSS searched the keywords for *HIV* and *AIDS*.

The following MeSH terms were included as exploded terms for *tuberculosis* if present in the database: Tuberculosis (Embase, PubMed, GH, CINAHL), Multidrug resistant tuberculosis (Embase), Tuberculosis, multidrug-resistant (PubMed, CINAHL), Latent tuberculosis (Embase), Infections Latent tuberculosis (PubMed), *Mycobacterium tuberculosis* (Embase, PubMed, GH, CINAHL), Drug resistant tuberculosis (Embase), Extensively drug resistant tuberculosis (Embase, PubMed). These were also combined with text word searches for the following terms: tuberculosis or TB or *Mycobacterium tuberculosis* or multi-drug resistant tuberculosis or multi-drug resistant TB or multiple drug resistant tuberculosis or multiple drug resistant TB or multidrug resistant tuberculosis or multidrug resistant TB or MDR tuberculosis or MDR TB or XDR TB or XDR tuberculosis or extensiv* drug resistant tuberculosis or extensiv* drug resistant TB. Searches in Scopus were performed on title and abstract for all terms, and as topics in Web of Science. IBSS searched the keyword *tuberculosis*.

The following MeSH terms were included as exploded terms for *prisoners* if present in the database: prison (Embase), prisons (PubMed), prisoner (Embase), prisoners (PubMed, GH, CINAHL), offender (Embase), correctional institutions (GH), correctional facilities (CINAHL). These were also combined with text word searches for the following: prison* OR inmate OR inmates OR jail OR gaol* OR correction* facilit* OR penitentiar* OR prison officer* OR penal institut* OR detention camp* OR custod* OR concentration camp* OR incarcerate* OR imprison* OR correctional setting* OR detain* OR detention* OR correctional centre. Searches in Scopus were performed on title and abstract for all terms, and as topics in Web of Science. IBSS searched the keyword *prison**.

The following MeSH term was included as an exploded term for *detention centres* if present in the database: detention camp (Embase). This was also combined with text word searches for the following: compulsory drug detention OR compulsory drug detention OR compulsory drug treatment OR compulsory rehabil* OR correction* center* OR correction* facilit* OR re-education through labour OR laojiaosuo OR long-term detention OR labour camp*. Searches in Scopus were performed on title and abstract for all terms, and as topics in Web of Science.

The prison and detention centre categories were combined with *OR*, following which all categories (HIV/AIDS, tuberculosis, prison/detention) were combined with *AND*, in all searches.

1800 of the 1975 papers reviewed failed to meet at least one of the inclusion criteria, most often due to a lack of specification and/or unacceptable diagnostic methods, or information only on co-infection policy, and were discarded. Therefore 175 papers remained for full appraisal for inclusion and the full text was retrieved.

For information on incidence and/or prevalence of co-infection, papers were discarded if published prior to 2000 or were published as a conference abstract only, due to the very different epidemiology of co-infection prior to 2000 and the limited peer review of conference abstracts. This excluded a further 48 papers, leaving 127/1975 papers. In addition, two papers could not be retrieved and 84 papers were excluded on full text review, leaving 41 papers for inclusion in regards to incidence/prevalence of co-infection (Figure 1). For information on prisoner co-infection outcomes, papers were not excluded solely on publication date. Of the 175 full text papers retrieved, 166 were excluded after a full text screen, leaving nine articles for inclusion in the review (Figure 2).

Thereafter 46/1975 papers were selected for inclusion in the review, 41 for incidence/prevalence estimates and nine for outcomes data, with four in both categories.

**Table A1:** Bias assessment of studies

Study	Diagnostic method	Case definition	Sample	Prevalent or incident	Response bias	Co-infection study	Loss to follow-up
CDC 2003	+	+	+	–	+	–	+
Aily 2013	+	+	–	–	?	+	+
Bosco de Oliveira 2004	?	+	+	–	?	–	–
Estevan 2013	–	–	–	–	–	–	–
Lemos 2009	+	–	–	–	+	–	+
Sanchez 2005	+	+	+	–	+	–	+
Valenca 2015	+	+	–	–	–	–	–
Noeske 2006	–	+	+	–	?	–	+
Noeske 2011	–	+	+	–	+	–	+
Rueda 2013	+	+	–	–	+	–	+
Rueda 2012	+	+	–	–	?	–	?
Gomez 2015	+	+	–	–	–	–	+
Moges 2012	–	+	–	–	+	–	+
Alavi 2014	?	+	–	–	–	–	+
Amwayi 2010	–	+	–	–	–	+	+
Margolis 2013	–	–	–	–	+	+	?
Al-Darraji 2013	–	+	–	–	+	–	+
Cerecer 2006	–	+	–	–	?	–	?
Hernandez-Leon 2012	–	+	–	+	?	–	?
Chigbu 2010	+	+	?	+	?	–	+
Lawal 2009	+	+	–	–	+	–	+
Drobniewski 2005	+	+	–	–	+	–	+
Telisinghe 2014	+	+	+	–	+	+	+
Davies 2012	?	+	–	–	+	–	+
Nyasulu 2015	?	+	–	–	?	–	+
Mnisi 2013	?	+	–	–	+	–	+
De la Hoz 2001	+	+	+	–	+	–	?
Fernandez-Martin 2000	+	+	–	–	?	–	?
Marco 2012	–	+	+	–	?	–	?
Martin 2001	+	+	+	+	?	–	–
Winetsky 2014	+	+	+	–	?	–	?
Rutta 2001	–	+	–	–	+	–	+
Schwitters 2014	?	+	–	–	+	–	+
Dubrovina 2008	–	+	–	–	+	–	+
Raykhert 2008	?	+	–	–	+	–	+
Ferenando 2014	+	+	+	–	+	+	+
MMWR 2007	?	+	?	–	?	–	?
Kim 2005	?	+	–	–	+	–	+
Lobato 2003	–	+	+	–	?	–	?
Baillargeon 2002	+	+	+	+	+	–	+
Henostroza 2013	+	+	+	–	?	+	?
Harris 2014	+	+	+	–	+	+	?
Maggard 2015	+	+	+	–	?	–	?

+ low risk of bias; ? unknown risk of bias; – high risk of bias. Diagnostic method: Risk of bias due to method used to confirm infection. Unknown diagnostics were studies based on known TB/HIV cases with no diagnostic method listed, often retrospective studies of hospital case notes. Case definition: Was a case definition clearly defined, for example active TB/LTBI/both? Sample: Was the sample representative of the prison as a whole, that is not within a prison subgroup only but a statistically representative sample? Prevalent or incident: Was only prevalence assessed, which may be open to bias, dependent on prison composition at this time point? Response bias: Was there any potential bias in those who refused TB/HIV screening? Co-infection study: Did the study specifically aim to measure co-infection? Loss to follow-up: Were many prisoners lost or results not read, for example from tuberculin skin test, due to prison turnover?

**Table A2:** Studies of HIV/TB co-infection, study design and summary measures

Author	Country	Prevalence of co-infection in whole prison population (%)	Prevalence of HIV in TB cases (%)	Prevalence of TB in HIV cases (%)	Measures of association	Incidence
CDC 2003	Botswana			30		
Aily 2013	Brazil	0.4	9 to 13			
Bosco de Oliveira 2004	Brazil		67.8			
Estevan 2013	Brazil		0.4		HIV positivity and LTBI, *p=*0.3618	
Lemos 2009	Brazil		0.0			
Sanchez 2005	Brazil		14.6		HIV positivity and TB, *p*≤0.0001	
Valenca 2015	Brazil		18.0		HIV positivity and TB, OR 1.57, 95% CI 0.47 to 6.48, *p*=0.397	
Noeske 2006	Cameroon		25.0			
Noeske 2011	Cameroon		10 to 15		HIV positivity and TB: *p*=0.83 new cases, *p*=0.003 prevalent cases	
Rueda 2013	Colombia		4.2			
Rueda 2012	Colombia		0.1			
Gomez 2015	Colombia		12.4			
Moges 2012	Ethiopia		73.1			
Alavi 2014	Iran		2.4		Co-infection compared to general population, *p*=0.025	
Amwayi 2010	Kenya				TB infection and HIV status, OR 10.75, 95% CI 2.42 to 47.77	
Margolis 2013	Malaysia			16.9	TB infection and HIV positivity, OR 1.82, 95% CI 0.88 to 3.76	
Al-Darraji 2013	Malaysia			16.7		
Cerecer 2006	Mexico		7.6			
Hernandez-Leon 2012	Mexico			16.3		7.7/100 persons/year active TB, 4.7/100 persons/year PTB
Chigbu 2010	Nigeria		0.6 (active), 14.9 (LTBI)			24% HIV-positive inmates with positive TST developed TB compared to 13.8% HIV negative
Lawal 2009	Nigeria		4.2			
Drobniewski 2005	Russia		12.2			
Telisinghe 2014	South Africa		25.3		HIV positivity and undiagnosed TB, OR 2.0, 95% CI 1.0 to 4.2	
Davies 2012	South Africa			56.1		
Nyasulu 2015	South Africa		58.0		HIV positivity and TB, OR 4.4, 95% CI 2.64 to 7.00	
Mnisi 2013	South Africa		54.0			
De la Hoz 2001	Spain		71.0		Current imprisonment and TB cluster HIV-negative cases, OR 6.3, 95% CI 1.8 to 21.8. Previous imprisonment and TB cluster HIV-positive cases, OR 13.0, 95% CI 3.7 to 45.6	
Fernandez-Martin 2000	Spain		71.0			
Marco 2012	Spain	6.7		63.2		
Martin 2001	Spain		10.0		TB and HIV positivity, OR 4.07, 95% CI 2.61 to 6.35	
Winetsky 2014	Tajikistan			33.3		
Rutta 2001	Tanzania		25.9			
Schwitters 2014	Uganda		57.1			
Dubrovina 2008	Ukraine		17.7			
Raykhert 2008	Ukraine		20.8			
Ferenando 2014	UK	0.0				
MMWR 2007	USA		16.0			
Kim 2005	USA		23.1		HIV status and TB, chi-square 39.58, *p*<0.01; HIV positivity and TB, OR 3.07, *p*<0.01	
Lobato 2003	USA		44.9			
Baillargeon 2002	USA					TB rate in HIV-positive prisoners 359/100,000 persons, 95% CI 214 to 725
Henostroza 2013	Zambia		35.7 to 50.0	6.4 to 8.3		
Harris 2014	Zambia		43.6		HIV positivity and TB, OR 2.4, 95% CI 1.5 to 4.1, *p*=0.0007	
Maggard 2015	Zambia		37.0			

CI, confidence interval; LTBI, latent tuberculosis infection; OR, odds ratio; TB, tuberculosis; TST, tuberculin skin testing; PTB, pulmonary tuberculosis.
